# MicroRNA-34a and MicroRNA-181a Mediate Visfatin-Induced Apoptosis and Oxidative Stress via NF-κB Pathway in Human Osteoarthritic Chondrocytes

**DOI:** 10.3390/cells8080874

**Published:** 2019-08-11

**Authors:** Sara Cheleschi, Sara Tenti, Nicola Mondanelli, Claudio Corallo, Marcella Barbarino, Stefano Giannotti, Ines Gallo, Antonio Giordano, Antonella Fioravanti

**Affiliations:** 1 Department of Medicine, Surgery and Neuroscience, Rheumatology Unit, Azienda Ospedaliera Universitaria Senese, Policlinico Le Scotte, 53100 Siena, Italy; 2 Department of Medicine, Surgery and Neurosciences, Section of Orthopedics and Traumatology, University of Siena, Policlinico Le Scotte, 53100 Siena, Italy; 3 Department of Medicine, Surgery and Neuroscience, Scleroderma Unit, University of Siena, Policlinico Le Scotte, 53100 Siena, Italy; 4 Sbarro Institute for Cancer Research and Molecular Medicine, Center for Biotechnology, College of Science and Technology, Temple University, Philadelphia, PA 19122, USA; 5 Department of Medical Biotechnologies, University of Siena, 53100 Siena, Italy

**Keywords:** visfatin, microRNA, osteoarthritis, apoptosis, oxidative stress, chondrocytes, miR-34a, miR-181a, NF-κB

## Abstract

Current evidence suggests a complex interaction between adipokines and microRNA (miRNA) in osteoarthritis (OA) pathogenesis. The present study explored the role of miR-34a and miR-181a in regulating apoptosis and oxidative stress induced by visfatin in human OA chondrocytes. Chondrocytes were transfected with miR-34a and miR-181a inhibitors and stimulated with visfatin for 24 h, in the presence of nuclear factor (NF)-κB inhibitor (BAY-11-7082, 2 h pre-incubation). Apoptosis and reactive oxygen species (ROS) production were detected by cytometry, miRNA, antioxidant enzymes, nuclear factor erythroid (NRF)2 and B-cell lymphoma (BCL)2 expressions by quantitative real time polymerase chain reaction (real time PCR) and western blot. P50 NF-κB subunit was measured by immunofluorescence. Visfatin significantly induced apoptosis and superoxide anion production, increased miR-34a, miR-181a, superoxide dismutase (SOD)-2, catalase (CAT), NRF2 and decreased BCL2 gene and protein expression in OA chondrocytes. All the visfatin-caused effects were suppressed by using miR-34a and miR-181a inhibitors. Pre-incubation with BAY-11-7082 counteracted visfatin-induced expression of miRNA, BCL2, SOD-2, CAT and NRF2. Inhibition of miR-34a and miR-181a significantly reduced the activation of p50 NF-κB. Visfatin confirms its ability to induce apoptosis and oxidative stress in human OA chondrocytes; these effects appeared mediated by miR-34a and miR-181a via NF-κB pathway. We highlight the relevance of visfatin as potential therapeutic target for OA treatment.

## 1. Introduction

MicroRNA (miRNA) are an abundant class of conserved non-coding RNA molecules of 22–25 nucleotides which emerged as important post-transcriptional regulators of gene expression either through the enhancement of degradation, by suppressing translation or via other mechanisms [1]. They have been associated with controlling important cellular processes or pathological features [1]. Several groups of investigators proposed that altered miRNA expression has been linked to regulation of cartilage homeostasis and osteoarthritis (OA) [2,3]. Furthermore, some miRNA were identified as oxidative stress-responsive miRNA [4], as well as, oxidative stress was found modulated by specific miRNA [5]. Thus, one of the possible mechanisms by which miRNA induce articular cartilage damage in OA could be represented by the variation of cellular redox state.

Obesity has been traditionally thought of as one of the most influential risk factors for OA [6,7]. White adipose tissue is an active endocrine organ producing multiple factors, most of them are of a pro-inflammatory nature such as classical cytokines, interleukin (IL)-6, IL-1β, tumour necrosis factor (TNF)-α and adipokines, adiponectin, leptin, resistin, chemerin and visfatin [6].

Adipokines are involved in various biological processes, including inflammation, immunity, cartilage and bone metabolism; their implications for the pathogenesis of many rheumatic diseases has been reported [6,8,9,10]. 

Among them, visfatin, pre-B cell colony-enhancing factor (PBEF) and nicotinamide phosphoribosyltransferase (Nampt), were originally identified as an insulin-mimetic factor, with pro-inflammatory and immunomodulating functions [10]. Serum visfatin levels were found increased in patients with OA [9,11]; moreover, some reports showed the significant pro-degradative effect of the adipokine in OA cartilage [12,13].

Recent research has highlighted possible cross-talk between miRNA and adipokines demonstrating the modulation by miRNA on adipocytes differentiation [14]; otherwise, adipokines that resulted were implicated in the regulation of some miRNA [12,13]. However, this complex and mutual interaction needs to be further elucidated.

In a previous study, in human OA chondrocytes, we evaluated the effect of visfatin on regulating apoptosis and the expression levels of a pattern of miRNA (miR-34a, miR-146a, miR-155, miR-181a, miR-140 and miR-let7e) and on the activation of nuclear factor (NF)-κB pathway. 

The present paper more deeply investigates the role of miR-34a and miR-181a in mediating visfatin-induced apoptosis and oxidative stress by transfection experiments with miRNA specific inhibitors.

In particular, we first analysed the effect of visfatin on regulating apoptosis percentage and the expression levels of apoptosis mediator B-cell lymphoma (BCL) 2, as well as the production of ROS (mitochondrial superoxide anion detection) and the mRNA levels of antioxidant enzymes [superoxide dismutase (SOD)-2, catalase (CAT)] and nuclear factor erythroid (NRF)2.

Furthermore, to examine the potential role of miR-34a and miR-181a as mediators of visfatin effects on apoptosis, oxidative stress and NF-κB activation we transfected chondrocytes with specific miRNA inhibitors.

Finally, the possible implication of NF-κB pathway in visfatin-mediated effects was also investigated. 

## 2. Materials and Methods

### 2.1. Chondrocyte Cultures

Human OA articular cartilage was obtained from femoral heads of five non-obese (BMI ranging from 20 to 23 kg/m^2^) and non-diabetic patients (two men and three women, age ranging from 67 to 77) with hip OA defined by the clinical and radiological ACR criteria [15], undergoing surgery for total hip replacement. OA grades ranged from moderate to severe and cartilage showed typical OA changes as the presence of chondrocyte clusters, fibrillation and loss of metachromasia (Mankin degree 3–7) [16]. OA chondrocytes were derived from the area adjacent to the OA lesion. The femoral heads were supplied by the Orthopaedic Surgery, University of Siena, Italy. The Ethics Committee of the Azienda Ospedaliera Universitaria Senese/Siena University Hospital approved the use of human articular samples (decision no. 13931/18) and all subjects signed an informed consent form.

Chondrocytes were isolated immediately after surgery. In brief, cartilage fragments were aseptically dissected from each donor and processed by an enzymatic digestion with trypsin (Sigma–Aldrich, Milan, Italy) for 15 min at 37 °C and then with type IV collagenase (Sigma–Aldrich, Milan, Italy) for 12–16 h at 37 °C.

The obtained cell suspension was filtered twice using 70-μm nylon meshes, washed and centrifuged for 5 min at 700× *g*. The viability was assessed by Trypan Blue (Sigma–Aldrich, Milan, Italy) test identifying 90% to 95% cell survival. Chondrocytes were recovered, seeded into 10-cm diameter tissue culture plates and were expanded for 10–12 days in a monolayer incubator with 5% CO_2_ and 90% humidified atmosphere at 37 °C until it reached 80% confluence.

Cells were grown in Dulbecco’s Modified Eagle Medium (DMEM) (Euroclone, Milan, Italy), containing 10% foetal bovine serum (FBS) (Euroclone, Milan, Italy), with 200 U/mL penicillin and 200 µg/mL streptomycin (P/S) (Sigma–Aldrich, Milan, Italy). The medium was changed every 2–3 days. The cell morphology was examined daily with an inverted microscope (Olympus IMT-2, Tokyo, Japan) and OA primary chondrocytes at the first passage were employed for the experiments to guarantee their phenotypic stability preserved which can occur when sub-cultured in monolayer [17]. For each single experiment a cell culture from a unique donor was used.

### 2.2. Treatment of Chondrocyte Cultures

Human OA chondrocytes, at the first passage, were plated in 6-well dishes at a starting density of 1 × 10^5^ cells/well until they became confluent. Human recombinant visfatin (Sigma–Aldrich, Milan, Italy) was first dissolved in phosphate buffered saline (PBS) (Euroclone, Milan, Italy), according to the manufacturer’s instructions and then it was diluted in the culture medium immediately before the treatment to reach the final concentration required.

The cells were cultured for 24 h in DMEM with 0.5% FBS and visfatin at concentration of 5 μg/mL and 10 μg/mL. The concentrations of the adipokine used in this in vitro study were selected according to those used by other authors [13,18]; the final concentrations were chosen based on the best results obtained in terms of viability.

Afterwards, cells were pre-incubated for 2 h with 1 μM BAY 11-7082 (NF-κB inhibitor, IKKα/β, Sigma–Aldrich, Milan, Italy) and then treated 24 h with the tested concentrations of visfatin. 

After the treatment, the media were removed, centrifuged and stored at −80 °C, while the cells were immediately processed to carry out flow cytometry analysis and quantitative real-time PCR.

### 2.3. Transfection of Chondrocytes

Cells were grown and maintained in 6-well dishes at a starting density of 1 × 10^5^ cells/well until they became 85% confluent in DMEM supplemented with 10% FBS; then the media were replaced with DMEM 0.5% FBS for 6 h before transfection. After this period, chondrocytes were transfected with miR-34a and miR-181a specific inhibitors (Qiagen, Hilden, Germany), at the concentration of 50 nM or with the negative controls siRNA (NC) (Qiagen, Hilden, Germany), at the concentration of 5 nM, in serum-free medium for 24 h. Media were removed and the cells were immediately collected or stimulated for 24 h with visfatin 5 μg/mL and 10 μg/mL. 

### 2.4. Detection of Apoptosis

The evaluation of apoptotic ratio was assessed by using Annexin V-FITC and propidium iodide (PI) (ThermoFisher Scientific, Milan, Italy) kit. OA chondrocyte were seeded in 12-well plates (8 × 10^4^ cells/well) in DMEM supplemented with 10% FBS for 24 h. Afterwards, the medium was removed and the cells cultured in DMEM with 0.5% FBS for the treatment. Then, chondrocytes were washed and harvested by trypsin, collected into cytometry tubes and centrifuged for 5 min at 700× *g*. After centrifugation procedure the pellet was resuspended in 100 μL of 1 X Annexin-binding buffer, 5 μL of Alexa Fluor 488 annexin-V conjugated to fluorescein (green fluorescence) and 1 μL of 100 μg/mL PI (red fluorescence) working solution. Annexin and PI were added to 100 μL of cell suspension. Chondrocytes were incubated 15 min at room temperature in the dark. Then, 600 μL of 1 × Annexin-binding buffer were added and the analysis at flow cytometry has been performed. A total of 10,000 events (1 × 10^4^ cells per assay) were measured by the instrument. The results were analysed with Cell Quest software (Version 4.0, Becton Dickinson, San Jose, CA, USA). The detection of apoptosis was assessed considering cells simultaneously stained with Alexa Fluor 488 annexin-V and PI; the analysis allows to discriminate viable cells (annexin-V and PI-negative), early apoptosis (annexin-V-positive and PI-negative) and late apoptosis (annexin-V and PI positives) [19].

The results were expressed as percentage of apoptotic cells (total apoptosis) and the data were expressed as mean of three independent experiments (mean ± standard deviation (SD).

### 2.5. RNA Isolation and Quantitative Real-Time PCR

Cells were grown and maintained in 6-well dishes at a starting density of 1 × 10^5^ cells/well until they became 85% confluent in DMEM supplemented with 10% FBS. Then, the medium was removed and the cells were cultured in DMEM with 0.5% FBS usually used during the treatment procedure.

Total RNA, including miRNA, was extracted using TriPure Isolation Reagent (Euroclone, Milan, Italy) according to the manufacturer’s instructions and was stored at −80 °C. The concentration, purity and integrity of RNA were evaluated by measuring the OD at 260 nm and the 260/280 and 260/230 ratios by Nanodrop-1000 (Celbio, Milan, Italy). The quality of RNA was verified by electrophoresis on agarose gel (FlashGel System, Lonza, Rockland, ME, USA). Reverse transcription for miRNA was carried out by the cDNA miScript PCR Reverse Transcription (Qiagen, Hilden, Germany), while the same procedure for target genes by QuantiTect Reverse Transcription Kit (Qiagen, Germany), according to the manufacturer’s instructions.

MiRNA and target genes were examined by real-time PCR using, miScript SYBR Green (Qiagen, Hilden, Germany) and QuantiFast SYBR Green PCR (Qiagen, Hilden, Germany) kits, respectively. 

A list of the used primers is reported in Table 1.

All qPCR reactions were achieved in glass capillaries with a LightCycler 1.0 (Roche Molecular Biochemicals, Mannheim, Germany) with LightCycler Software Version 3.5. The reaction procedure for miRNA consisted of 95 °C for 15 min for HotStart polymerase activation, followed by 40 cycles of 15 s at 95 °C for denaturation, 30 s at 55 °C for annealing and 30 s at 70 °C for elongation, according to the protocol. Target gene amplification was performed at 5 in at 95 °C, 40 cycles of 15 s at 95 °C and 30 s at 60 °C. In the final step of both protocols, the temperature was raised from 60 °C to 95 °C at 0.1 °C/step to plot the melting curve.

To analyse the dissociation curves, we visualized the amplicons lengths in agarose gel to confirm the correct amplification of the resulting PCR products.

For the data analysis, the *C*_t_ values of each sample and the efficiency of the primer set were calculated with LinReg Software and were then converted into relative quantities and normalized.

The normalization was performed considering Small Nucleolar RNA, C/D Box 25 (SNORD-25) for miRNA and Actin Beta (ACTB) for target genes, as the housekeeping genes. These genes were chosen according to geNorm software version 3.5.

### 2.6. Mitochondrial Superoxide Anion (•O_2_^−^) Production

OA chondrocyte were seeded in 12 well-plates (8 × 10^4^ cells/well) in DMEM supplemented with 10% FBS for 24 h. After this period, the medium was discarded and the cells were cultured in DMEM with 0.5% FBS for the treatment. Then, the chondrocytes were incubated in Hanks’ Balanced Salt Solution (HBSS) and MitoSOX Red for 15 min at 37 °C in the dark, to assess mitochondrial superoxide anion (•O_2_^−^) production. MitoSOX probe was dissolved in DMSO to reach the final concentration required of 5 µM. Cells were then harvested by trypsin and collected into cytometry tubes and centrifuged at 700× *g* for 5 min. After centrifugation step, pellets were resuspended in saline solution before analysis at flow cytometry. A density of 1 × 10^4^ cells per assay (a total of 10,000 events) were measured by the instrument and data were analysed with CellQuest software (Version 4.0, Becton Dickinson, San Jose, CA, USA). Results were expressed as median of fluorescence (AU) and indicated as mean of three independent experiments (mean ± SD).

### 2.7. Western Blot

OA chondrocytes at first passage were seeded in Petri dishes (35 × 10 mm) at a starting density of 1 × 10^5^ cells/chamber in DMEM supplemented with 10% FBS for 24 h. After this period, the medium was removed and the cells were cultured in DMEM with 0.5% FBS for the experiment. After treatment, samples were collected and total cell lysates were obtained with M-PER™ Mammalian Protein Extraction Reagent (Thermo-Fisher Scientific, Rockford, IL, USA) containing a protease inhibitor cocktail (Sigma-Aldrich S.r.l., Milan, Italy). Protein concentration was determined by the Bradford method (Bio-Rad Laboratories S.r.l., Milan, Italy). For each experimental condition ten micrograms were loaded into 10% sodium dodecyl sulphate-polyacrylamide electrophoresis gels and separated by molecular size. Proteins were then transferred to a nitrocellulose membrane and, after blocking step, incubated at 4 °C overnight with mouse monoclonal anti-BCL2 (sc-509), anti-SOD-2 (sc-130345), anti-CAT (sc-271358) and NRF2 (sc-81342) (Santa Cruz Biotechnology, Italy). Afterwards, the samples were incubated with secondary goat anti-mouse IgG (H+L)-HRP conjugate antibody (1:5000) (Bio-Rad Laboratories S.r.l., Milan, Italy); the reaction was assessed by chemiluminescence (Bio-Rad Laboratories S.r.l., Milan, Italy). The blots were re-probed with HRP-conjugated β-actin (Sigma-Aldrich S.r.l., Milan, Italy) used as the loading control. Images of the bands were digitized and the densitometric quantification of the bands was performed by Image-J software (LOCI, University of Wisconsin-Madison, Madison, WI, USA). Results were normalized with the relative loading control.

### 2.8. Immunofluorescence Analysis

Human OA chondrocytes were plated in coverslips in Petri dishes (35 × 10 mm) at a starting low density of 4 × 10^4^ cells/chamber, to prevent possible cell overlapping and re-suspended in DMEM supplemented with 10% FBS for 24 h until 80% of confluence. The cells were processed after 24 h of transfection with miR-34a and miR-181a specific inhibitors to evaluate the potential activation of the NF-κB pathway. Chondrocytes were washed in PBS and then fixed in 4% paraformaldehyde (ThermoFisher Scientific, Milan, Italy) (pH 7.4) for 15 min at room temperature. Afterwards, the cells were permeabilized with a blocking solution, constituted by PBS, 1% bovine serum albumin (BSA) (Sigma–Aldrich, Milan, Italy) and 0.2% Triton X-100 (ThermoFisher Scientific, Milan, Italy), for 20 min at room temperature. Then, chondrocytes were incubated overnight at 4 °C with mouse monoclonal anti-p50 subunit primary antibody (sc-8414, Santa Cruz Biotechnology, Italy) (dilution 1:100) dissolved in PBS, 1% BSA and 0.05% Triton X-100. After this period, coverslips were washed three times in PBS and incubated for 1 h with goat anti-mouse IgG-Texas Red conjugated antibody (Southern Biotechnology, Italy) (dilution 1:100) dissolved in PBS, 1% BSA and 0.05% Triton X-100. Finally, after three washes in PBS the coverslips were submitted to nuclear counterstain by 4,6-diamidino-2-phenylindole (DAPI) and then mounted with Vecta shield (Vector Labs). Fluorescence was examined under an Zeiss Axiovert (Zeiss, Germany) light microscope equipped with epifluorescence at 200× and 400× magnification. Immunoreactivity of p50 was semi-quantified as mean densitometric area of p50 signal at nuclear and cytoplasmic level, by AxioVision 4.6 software measure program [17]. At least 100 chondrocytes from each group were analysed.

### 2.9. Statistical Analysis

Three independent experiments were carried out and the results were expressed as the mean ± SD of triplicate values for each experiment. Data normal distribution was evaluated by Shapiro–Wilk, D’Agostino and Pearson and Kolmogorov–Smirnov tests.

Flow cytometry and western blot results were analysed by ANOVA with Bonferroni post-hoc test. Quantitative real-time PCR data were evaluated by one-way ANOVA with a Tukey’s post-hoc test using 2^−ΔΔCT^ values for each sample.

All analyses were performed through the SAS System (SAS Institute Inc., Cary, NC, USA) and GraphPad Prism 6.1. A *p*-value < 0.05 was defined as statistically significant.

## 3. Results

### 3.1. Visfatin Regulates Apoptosis and Oxidative Stress Processes

**Figure 1:**
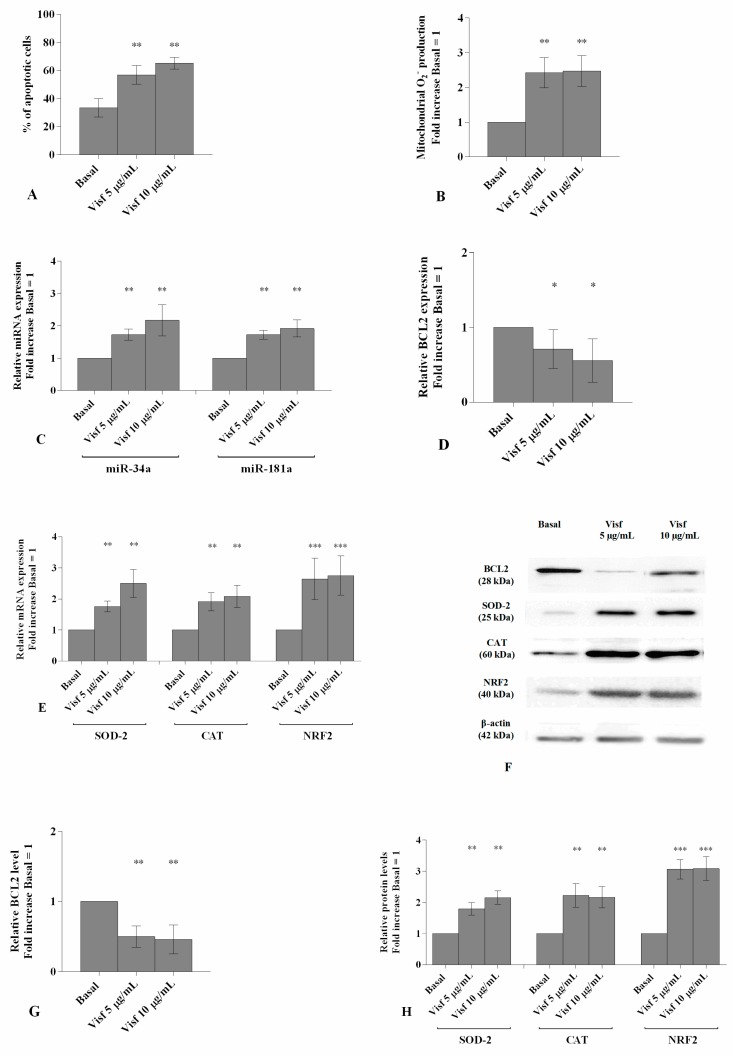
(**A**) Detection of apoptosis by flow cytometry analysis. Apoptosis was measured with Annexin Alexa fluor 488 assay. Data were expressed as percentage of positive cells for Annexin-V and propidium iodide (PI) in all the studied conditions. (**B**) The production levels of mitochondrial superoxide anion by flow cytometry analysis using MitoSox Red staining. (**C**–**E**) Expression levels of miR-34a, miR-181a, B-cell lymphoma (BCL2), superoxide dismutase (SOD-2), catalase (CAT), nuclear factor erythroid 2 like 2 (NRF2) by real-time PCR. (**F**–**H**) Representative immunoblotting image and densitometric analysis of BCL2, SOD-2, CAT, NRF2 protein levels by western blot. Human osteoarthritic (OA) chondrocytes were evaluated at basal condition and after 24 h of stimulus with visfatin (5 μg/mL and 10 μg/mL). The production of superoxide anion, the gene expression and the protein levels were referenced to the ratio of the value of interest and basal condition (Basal, cells without treatment). The value of basal condition was reported equal to 1. Data were expressed as mean ± SD of triplicate values. * *p* <0.05, ** *p* < 0.01, *** *p* < 0.001 versus basal condition. Visf = Visfatin.

The stimulus of OA chondrocytes with visfatin at concentration of 5 μg/mL and 10 μg/mL determined a significant induction of apoptosis percentage (*p* < 0.01, Figure 1A) and an increase of superoxide anion production (*p* < 0.01, Figure 1B) in comparison to basal condition.

Furthermore, visfatin significantly increased the gene expression of miR-34a and miR-181a (*p* < 0.01, Figure 1C) and the mRNA and protein levels of antioxidant enzymes SOD-2 (*p* < 0.01), CAT (*p* < 0.01) and NRF2 (*p* < 0.001) (Figure 1E,F,H).

The gene and protein expression of BCL2 resulted decreased, in a significant manner, by both tested concentrations of visfatin (*p* < 0.05, *p* < 0.01, Figure 1D,F,G) when compared to baseline.

### 3.2. MiRNA Specific Inhibitors Block Visfatin Effect on Mir-34a and Mir-181a Gene Expression

**Figure 2:**
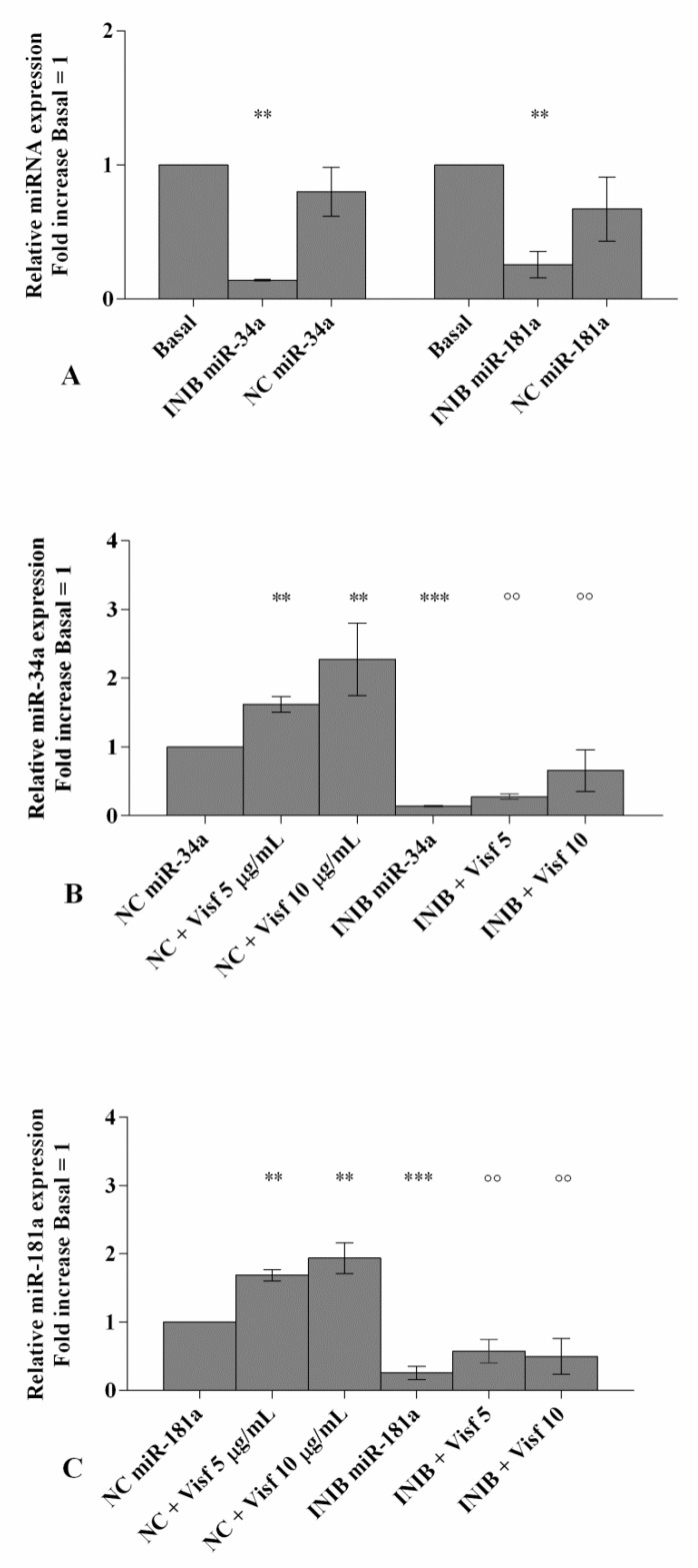
(**A**–**C**) Expression levels of miR-34a and miR-181a by real-time PCR. Human osteoarthritic (OA) chondrocytes were evaluated at basal condition, after 24 h of transfection with miR-34a and miR-181a inhibitors (50 nM) or NC (5 nM) and after 24 h of stimulus with visfatin (5 μg/mL and 10 μg/mL). The gene expression was referenced to the ratio of the value of interest and basal condition (Basal, cells without treatment) or NC. The value of basal condition or NC were reported equal to 1. Data were expressed as mean ± SD of triplicate values. ** *p* < 0.01, *** *p* < 0.001 versus basal condition or NC. °° *p* < 0.01 versus inhibitor. INIB = Inhibitor, NC = negative control siRNA, Visf = Visfatin.

To confirm whether the expression of miR-34a and miR-181a was induced by visfatin stimulus, miR-34a and miR-181a inhibitors were transfected into OA chondrocytes (Figure 2). 

Real-time PCR revealed that miRNA inhibitors significantly reduced the gene expression of miR-34a and miR-181a (*p* < 0.01) when compared with basal condition and NC (Figure 2A). 

Visfatin 5 μg/mL and 10 μg/mL significantly up-regulated mRNA levels of the studied miRNA (*p* < 0.01, Figure 2B,C) in chondrocytes transfected with NC. After the transfection with miRNA inhibitors, OA chondrocytes stimulated with visfatin did not show any significant modification in miR-34a and miR-181a gene expression with respect to the cells transfected with the specific inhibitors alone (Figure 2B,C). Moreover, miR-34a and miR-181a inhibitors significantly inhibited the increase of miRNA expression levels induced by visfatin stimulus (*p* < 0.01, Figure 2B,C).

### 3.3. MiR-34a and Mir-181a Specific Inhibitors Prevent Chondrocyte Apoptosis Induced by Visfatin by Regulating BCL2

**Figure 3:**
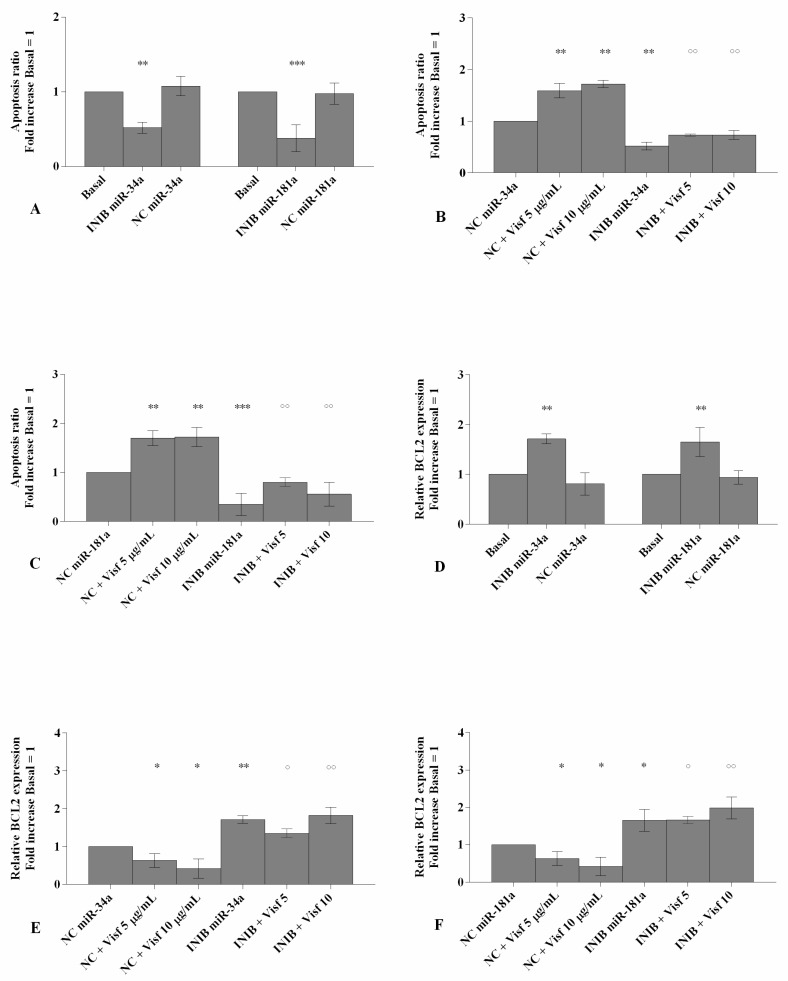
(**A**–**C**) Detection of apoptosis by flow cytometry analysis. Apoptosis was measured with Annexin Alexa fluor 488 assay. Data were expressed as percentage of positive cells for Annexin-V and propidium iodide (PI) in all the studied conditions. (**D**–**F**) Expression levels of B-cell lymphoma (BCL2) by real-time PCR. Human osteoarthritic (OA) chondrocytes were evaluated at basal condition, after 24 h of transfection with miR-34a and miR-181a inhibitors (50 nM) or NC (5 nM) and after 24 h of stimulus with visfatin (5 μg/mL and 10 μg/mL). The ratio of apoptosis and the gene expression were referenced to the ratio of the value of interest and basal condition (Basal, cells without treatment) or NC. The value of basal condition or NC were reported equal to 1. Data were expressed as mean ± SD of triplicate values. *p < 0.05, **p < 0.01, ***p < 0.001 versus basal condition or NC. ° *p* < 0.05, °° *p* < 0.01 versus inhibitor. INIB = Inhibitor, NC = negative control siRNA, Visf = Visfatin.

Figure 3 reported the modulation of apoptosis, induced by visfatin, after the transfection of OA chondrocytes with miR-34a and miR-181a specific inhibitors. 

Cells transfected with miR-34a and miR-181a inhibitors showed a significant reduction of apoptosis ratio (*p* < 0.01, *p* < 0.001, Figure 3A) and an up-regulation of BCL2 expression levels (*p* < 0.01, Figure 3D) in comparison to basal condition and NC.

The stimulus with visfatin at the concentration of 5 μg/mL and 10 μg/mL significantly raises the amount of apoptotic cells (*p* < 0.01) and reduced the expression levels of BCL2 (*p* < 0.05) in chondrocytes transfected with NC when compared to baseline (Figure 3B,C,E,F). On the contrary, after the incubation with miR-34a and miR-181a inhibitors, no changes on apoptosis and on BCL2 gene levels in visfatin-stimulated OA cells were observed with respect to those induced by the effect of specific inhibitors alone (Figure 3B,C,E,F). 

The effect of visfatin on apoptosis ratio and on BCL2 expression levels was inhibited, in a significant manner, by miR-34a and miR-181a specific inhibitors (*p* < 0.05, *p* < 0.01, Figure 3B,C,E,F). 

### 3.4. MiR-34a and MiR-181a Specific Inhibitors Regulate Oxidative Stress Induced by Visfatin

**Figure 4:**
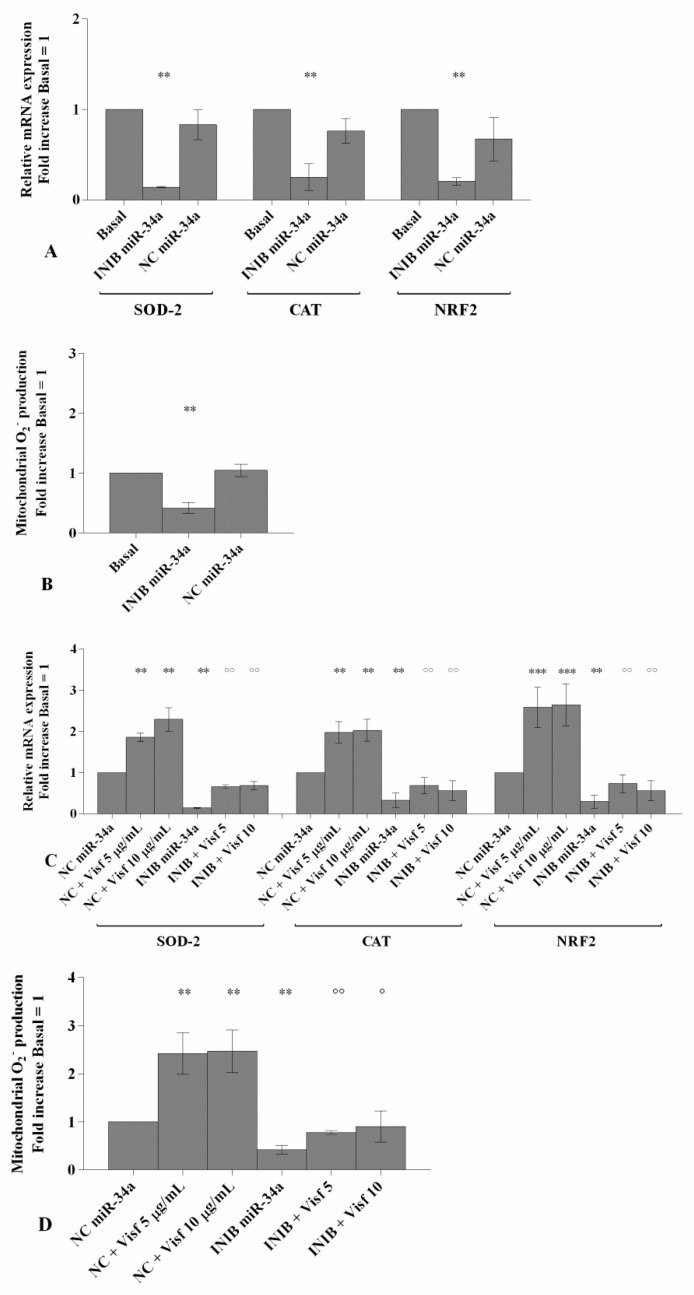

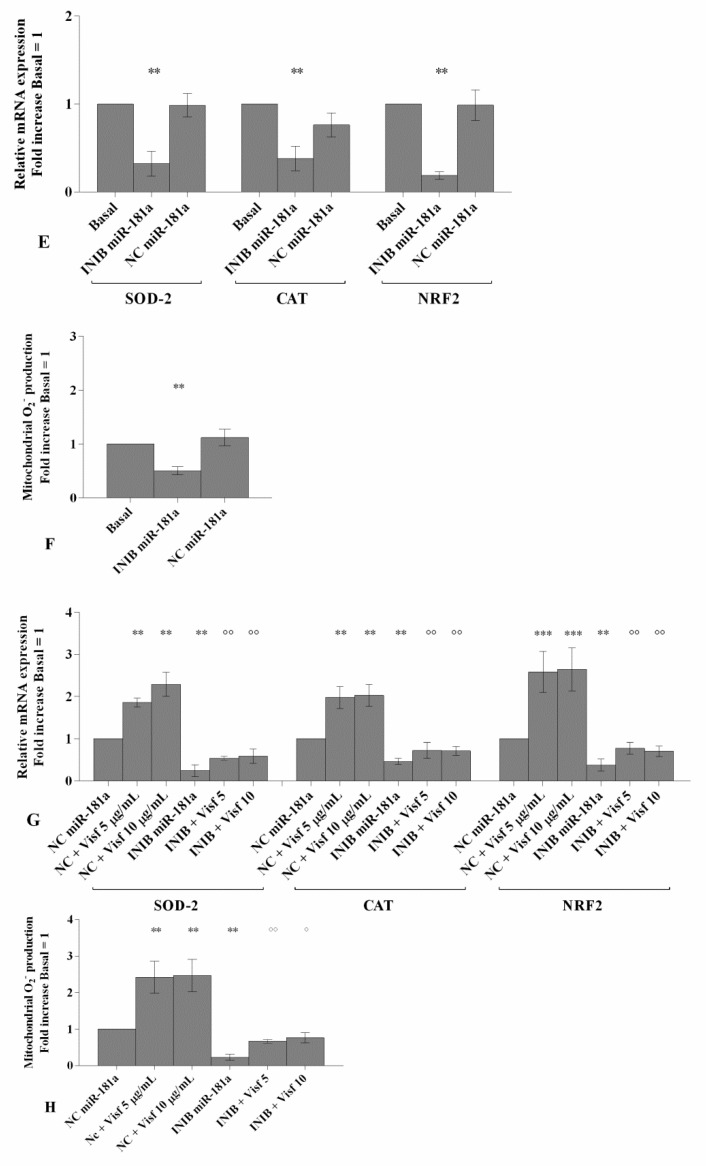
(**A**,**C**,**E**,**G**) Expression levels of superoxide dismutase (SOD-2), catalase (CAT), nuclear factor erythroid 2 like 2 (NRF2) by real-time PCR. (**B**,**D**,**F**,**H**) The production levels of mitochondrial superoxide anion by flow cytometry analysis using MitoSox Red staining. Human osteoarthritic (OA) chondrocytes were evaluated at basal condition, after 24 h of transfection with miR-34a and miR-181a inhibitors (50 nM) or NC (5 nM) and after 24 h of stimulus with visfatin (5 μg/mL and 10 μg/mL). The gene expression and the production of superoxide anion were referenced to the ratio of the value of interest and basal condition (Basal, cells without treatment) or NC. The value of basal condition or NC were reported equal to 1. Data were expressed as mean ± SD of triplicate values. ** *p* < 0.01, *** *p* < 0.001 versus basal condition or NC. ° *p* < 0.05, °° *p* < 0.01 versus inhibitor. INIB = Inhibitor, NC = negative control siRNA, Visf = Visfatin.

Figure 4 highlights the regulation of oxidant/antioxidant balance, induced by visfatin, after the transfection of OA chondrocytes with miR-34a and miR-181a inhibitors. 

Chondrocytes transfected with miR-34a and miR-181a inhibitors showed a significant down-regulation of SOD-2, CAT and NRF2 expression levels (*p* < 0.01, Figure 4A,E) as well as of mitochondrial superoxide anion production (*p* < 0.01, Figure 4B,F) in comparison to basal condition and NC. The expression levels of SOD-2, CAT, NRF2 and the production of superoxide anion were significantly increased after incubation with visfatin (*p* < 0.01, Figure 4C,D,G,H) in OA chondrocytes transfected with NC. The effect of visfatin on antioxidant enzymes expression levels and on superoxide anion production was significantly inhibited by the use of miR-34a and miR-181a specific inhibitors (*p* < 0.05, *p* < 0.01, Figure 4C,D,G,H).

### 3.5. NF-κB Pathway Mediates Visfatin Effects 

**Figure 5:**
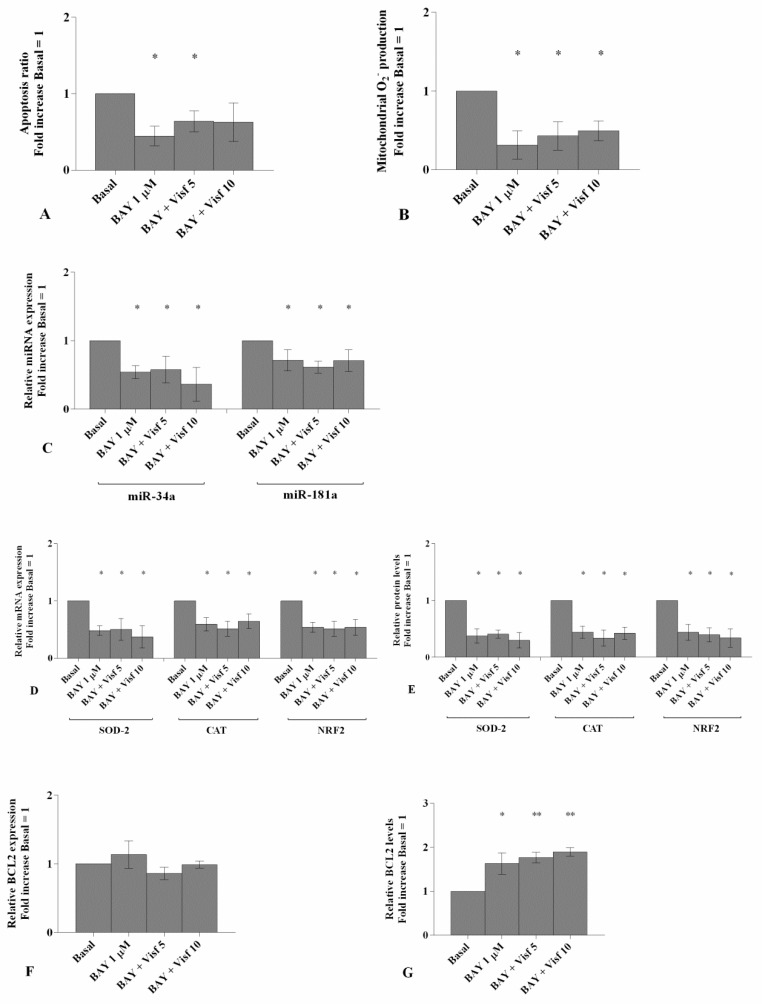
(**A**) Detection of apoptosis by flow cytometry analysis. Apoptosis was measured with Annexin Alexa fluor 488 assay. Data were expressed as percentage of positive cells for Annexin-V and propidium iodide (PI) in all the studied conditions. (**B**) The production levels of mitochondrial superoxide anion by flow cytometry analysis using MitoSox Red staining. Expression levels of miR-34a, miR-181a (**C**), superoxide dismutase (SOD-2), catalase (CAT), nuclear factor erythroid 2 like 2 (NRF2) (**D**) and B-cell lymphoma (BCL2) (**F**) by real-time PCR. (**E**,**G**) Densitometric analysis of SOD-2, CAT, NRF2 and BCL2 protein levels by western blot. Human osteoarthritic (OA) chondrocytes were evaluated at basal condition, after 2 h pre-incubation with a specific nuclear factor (NF)-κB inhibitor (BAY 11-7082, IKKα/β, 1 μM) and after 24 h of stimulus with visfatin (5 μg/mL and 10 μg/mL). The ratio of apoptosis, the production of superoxide anion, the gene expression and the protein levels were referenced to the ratio of the value of interest and basal condition (Basal, cells without treatment). The value of basal condition was reported equal to 1. Data were expressed as mean ± SD of triplicate values. * *p* < 0.05, ** *p* < 0.01 versus basal condition. BAY = BAY 11-7082, Visf = Visfatin.

The role of the NF-κB pathway in visfatin-based effects was summarized in Figure 5. 

The analysis of flow cytometry showed a significant decrease of apoptosis ratio and of superoxide anion production (*p* < 0.05, Figure 5A,B) in OA chondrocytes treated with a specific NF-κB inhibitor (IKKα/β, BAY 11-7082), alone or co-incubated with visfatin, in comparison to basal condition.

The gene expression of miR-34a and miR-181a resulted significantly down-regulated (*p* < 0.05, Figure 5C) as well as the mRNA and protein levels of the target genes SOD-2, CAT and NRF2 (*p* < 0.05, Figure 5D,E) in OA cells incubated with NF-κB inhibitor with or without visfatin, when compared to basal state.

A significant increase of BCL2 protein expression (*p* < 0.05, *p* < 0.01, Figure 5G) was found in OA chondrocytes treated with NF-κB inhibitor alone or in presence of visfatin, while no modifications in its gene levels were observed (Figure 5F). 

Cells co-treated with BAY 11-7082 plus visfatin did not exhibit any difference in miRNA and target genes expression to those incubated with BAY 11-7082 alone (Figure 5A–G).

### 3.6. MiR-34a and MiR-181a Regulate NF-κB Pathway

**Figure 6:**
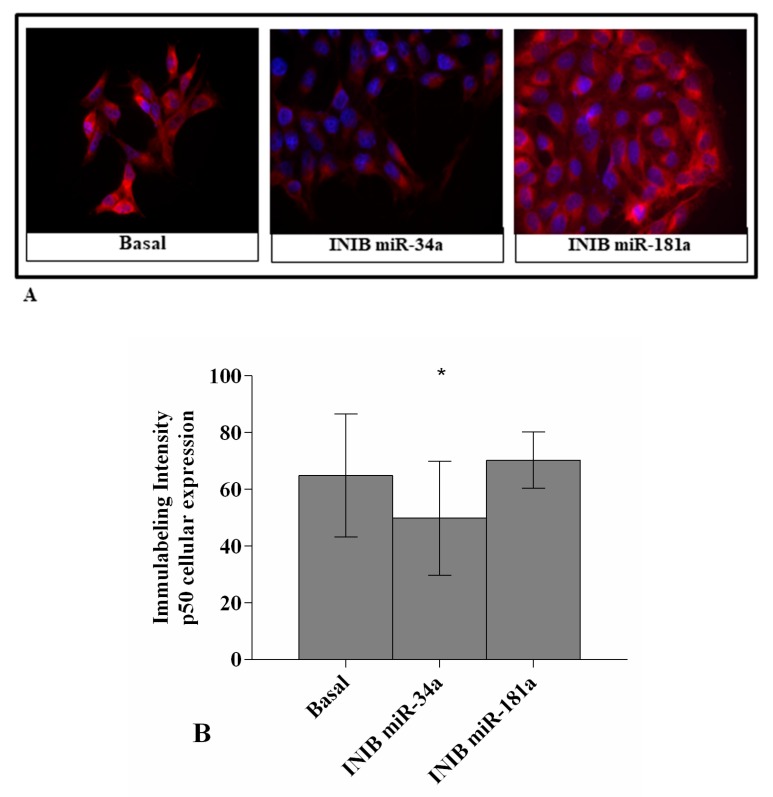
Immunofluorescence labelling of p50 NF-κB subunit localization. Human osteoarthritic (OA) chondrocytes were evaluated at basal condition and after 24 h of transfection with miR-34a and miR-181a specific inhibitors (50 nM). (**A**) Representative immunocytochemical images of the cells showing localization of p50 NF-κB (red); nuclei were stained with DAPI (blue). Original Magnification 400×. Scale bar: 20 μm. (**B**) The histogram of immunolabeling intensity was plotted for the nuclear and cytoplasm expression for p50 subunit. Data were expressed as mean ± SD of triplicate values. * *p* < 0.05 versus basal condition. INIB = Inhibitor.

Figure 6A,B showed the activation and the nuclear translocation of p50 NF-κB subunit in OA chondrocytes transfected with miR-34a and miR-181a specific inhibitors.

The signal of p50 subunit was consistently detected in the cytoplasm of chondrocytes at basal condition. A significant decrease of p50 cytoplasmic levels and nuclear translocation was observed in OA cells transfected with miR-34a inhibitor (*p* < 0.05), whit a resulting lower immunolabeling intensity, when compared with baseline. 

No detectable changes of p50 subunit activation and translocation in miR-181a inhibited-OA chondrocytes were reported.

## 4. Discussion

As a chronic degenerative joint disorder, OA represents one of the major contributor of disability and impairment in adult and elderly populations. Its features resulting in a progressive degeneration of articular cartilage, low-grade synovitis, leading to the loss of normal joint structure and function [20]. 

Current evidence highlights a critical role of adipokines and miRNA in the development and in the progression of OA, even if controversial results about their exact impact on cartilage metabolism were described [3,6,7,13]. 

A growing number of studies suggest a complex and mutual interaction between adipokines and miRNA [13,21,22,23]. To this purpose, in a previous paper, we characterized the expression profile of some miRNA in human OA chondrocytes incubated with visfatin and resistin, showing the modulation of the analysed miRNA by these adipokines via the NF-κB signalling pathway [13]. In the present study, we hypothesized that silencing miR-34a and miR-181a, by specific inhibitors, visfatin-induced apoptosis and oxidative stress, through the regulation of NF-κB pathway, could be counteracted.

Apoptosis is a highly regulated form of programmed cell death that during normal development constitutes a physiological sequence of events leading to the elimination of old, unnecessary and unhealthy cells. The dysregulation of apoptosis leads to pathological conditions, including cancer, autoimmune diseases or degenerative diseases, such as OA [24]. Apoptosis can be induced by mitochondrion dysfunction through an imbalance in release of pro- and anti-apoptotic proteins in the cytosol. BCL-2 family proteins are known as anti-apoptotic factors playing a pivotal role in the regulation of apoptotic processes; its over-expression protects OA chondrocytes from the programmed cell death [24]. 

In the current research we demonstrated the ability of visfatin to increase the percentage of apoptotic cells and to reduce the gene expression and protein levels of BCL2 in human OA chondrocytes cultures. Our results are consistent with what observed by other Authors that showed the regulation of apoptosis and of BCL2 protein after the stimulus of endothelial cells and chondrocytes with this adipokine [13,18,25]. Our results confirm the pro-apoptotic effect of visfatin in human OA chondrocytes cultures.

A well-known inducer of apoptosis signalling is miR-34a [26], which is widely expressed in different species and tissues and results as an anti-proliferative factor playing a role in the regulation of cell cycle arrest or senescence [27]. Its abnormal expression is correlated with several human diseases and its contribution in the development and in the progression of OA was demonstrated [26]. Besides, miR-34a caused apoptosis and limited cell proliferation of OA chondrocytes through direct regulation of the sirtuin (SIRT)-1/p53 signalling pathway [26]. 

Mir-181a, first associated with the maturation and the activation of immune cells, has been related to the regulation of inflammatory processes in autoimmune diseases and in OA [28]. This miRNA was found at higher levels in circulating PBMC of OA patients and it was correlated with severity of OA [29]; furthermore, studies on human OA facet cartilage and chondrocyte cultures showed its increased gene expression levels when compared to normal samples [30,31,32].

MiR-181a results also implicated in the regulation of apoptosis signalling by targeting multiple anti-apoptotic BCL2 family members in degenerative and autoimmune disorders [33,34].

The over-expression of miR-34a and miR-181a was found in OA chondrocytes stimulated by IL-1β and TNF-α [35,36]. Similarly, in our study visfatin, at the tested concentrations, significantly increased the expression levels of miR-34a and miR-181a in OA cells. Therefore, we can speculate that the negative effect of visfatin on apoptosis regulation could be mediated by the studied miRNA.

Transfection experiments on human primary OA and C-28/I2 chondrocyte cultures showed the direct targeting of miR-34a, miR-181a and miR-9 on SIRT-1/p53 and on BCL2 signalling pathways responsible for the regulation of chondrocyte proliferation and apoptosis during OA damage [26,37,38]. Our data are in agreement with these results demonstrating that specific inhibitors of miR-34a and miR-181a significantly reduced chondrocytes apoptosis and increased expression of BCL2 gene; moreover, transfection of the cells counteracted the apoptotic process induced by visfatin through targeting BCL2 protein. To the best of our knowledge, this is the first study revealing the direct correlation between miR-34a, miR-181a and visfatin in modulating apoptosis signalling.

Oxidative stress is one of the most important mechanisms underlying OA; the failure in oxidant/antioxidant balance in OA chondrocytes determines an altered redox status which promotes cartilage breakdown and makes cells more susceptible to oxidant-mediated apoptosis, contributing to the damage that occur in OA condition [4,5,39].

In our study, the analysis of endogenous production of ROS showed increased levels of mitochondrial superoxide anion content in OA chondrocytes stimulated with visfatin. No previous researches were performed to investigate the role of visfatin in oxidative stress induction in chondrocytes, however, Teixeira et al. [40] showed an increase of superoxide anion production after the stimulus of HUVEC lines with leptin.

The excessive production of endogenous ROS is balanced by the antioxidant defence system including enzymes such as SOD-2, CAT and glutathione peroxidase (GPx). SOD-2 or manganese-dependent (Mn)-SOD is located in the mitochondrial matrix of the cells and catalyses the dismutation of superoxide anion in O_2_ and H_2_O_2_, which is then turned, in water, to CAT [39]. Some reports showed a modification of these stress-related enzymes expression in OA chondrocytes, leading to the loss of their ability to scavenge ROS and contributing to cartilage breakdown in OA [4,39,41]. 

No studies have been carried out about the modulation of antioxidant enzymes in visfatin-stimulated cell cultures. We demonstrated the up-regulation of SOD-2 and CAT gene and protein expressions induced by visfatin in OA chondrocytes. Similarly, recent evidence reported an increase of SOD-2 and CAT mRNA and protein levels following the stimulus of the pro-inflammatory cytokines IL-1β and IL-6 in bovine normal and human OA chondrocytes cultures [19,42].

The rapid increase of detoxificant factors in visfatin-stimulated chondrocytes could be interpreted as an acute adaptive response to the over-expression of mitochondrial superoxide anion content induced by the adipokine, to protect mitochondria from the deleterious effects of oxidant agents. Indeed, the accumulation of ROS species, such as superoxide anion, impairs the normal function of mitochondria; ROS enhance mitochondrial DNA deletion, inactivate electron transport processes and then induce energy depletion and progressive cell death [43].

Besides, the excessive amount of ROS increases free radical scavenging activity, through the ability of the cells to enhance the endogenous defence system by inducing antioxidant/detoxifying enzymes through activation of transcription factors such as Nrf2 [42].

It is widely accepted that NRF2 is an important regulator in the homeostasis of the cellular redox state since its target genes include SOD-2, CAT and GPx. Stress signals lead to its accumulation and translocation into the nucleus, where it regulates the transcription of antioxidant response element-dependent genes [44]. The activation of NRF2 activity seems to represent a possible therapeutic approach for OA taking into account its ability to suppress metalloproteinases expression induced by IL-1β and regulating apoptotic cell death and oxidative stress processes [45]. 

Our results reported the over-expression of NRF2 mRNA and protein levels in OA chondrocytes stimulated with visfatin. A similar effect on NRF2 factor was observed by Teixeira et al. [40] in leptin-stimulated HUVEC lines.

These data first showed the involvement of visfatin in regulating redox balance and in controlling NRF2 activity.

The gene expression of miR-34a and miR-181a is induced by the oxidative stress stimulus as demonstrated in different studies from human cardiac and carcinoma cell lines and in OA chondrocytes [4,46,47]. Moreover, the inhibition of miR-34a and of miR-181a decreased the expression of antioxidant enzymes with a concomitant reduction of mitochondrial intracellular ROS levels in primary mesangial cells, in PBMC of Holstein cows and in HUVEC lines [48,49]. According to the current literature, transfection of our OA cells with miR-34a and miR-181a specific inhibitors significantly down-regulated the expression of antioxidant enzymes as well as the production of mitochondrial superoxide anion, particularly preventing their induction by visfatin. Similarly, Wu et al. [12] showed the inhibition of visfatin activity after transfection of synovial fibroblasts with miR-199 mimic.

These data point out the responsiveness of miR-34a and miR-181a to visfatin stimulus and their involvement in the regulation of oxidant/antioxidant balance; this confirm the complex interaction between miRNA and visfatin in OA pathogenesis.

We more deeply investigated the involvement of NF-κB signalling pathway in the relationship between visfatin and miRNA in the modulation of cartilage catabolism.

The role of NF-κB signalling in inflammatory and degrading processes of OA [50,51] is well known, as well as its activation and translocation caused by visfatin in studies from different cell types, including our previous paper [13,25,52]. Besides, some reports reveal the direct implication of NF-κB pathway in visfatin-mediated effects in human OA chondrocytes and in endothelial progenitor cells incubated with a specific NF-κB inhibitor [13,25,52]. In particular, these studies demonstrated the involvement of NF-κB in regulating apoptosis and expression levels of matrix degrading enzymes and pro-inflammatory factors induced by visfatin [13,25,52].

Furthermore, recent evidence highlights that a number of miRNA inhibits NF-κB activation and the production of the downstream genes regulated by the signalling pathway [53,54,55]. Transfection of murine macrophages with miR-210 specific inhibitor increased LPS-induced expression of some pro-inflammatory cytokines, by targeting NF-κB1 and decreasing NF-κB activation [53]. Wen et al. [54] found that the use of miR-145 mimic or inhibitor was able to regulate glucose uptake and insulin resistance in HepG2 cells stimulated with resistin, via p65 pathway. More recently, a study performed on human OA chondrocytes transfected with miR-27 mimic or inhibitor underlined the modulatory activity of this miRNA in inflammatory and degrading processes, that occur in OA pathogenesis, by regulating NF-κB pathway [55].

In agreement with these findings, we showed that the incubation of OA chondrocytes with NF-κB specific inhibitor reversed the effect of visfatin on the apoptosis and oxidative stress, on the gene expression of miR-34a and miR-181a and on the mRNA and protein levels of antioxidant enzymes (SOD-2, CAT, NRF2) and BCL2. We also proved that the silencing of miR-34a and miR-181a limited NF-κB translocation into the nucleus in OA chondrocytes cultures, suggesting the implication of the studied miRNA in mediating NF-κB pathway activity.

Taken together, these results indicate that visfatin is effective on chondrocyte apoptosis and oxidative stress processes through the regulation of NF-κB signalling pathway. The reduction of visfatin effects, through NF-κB modulation, were observed after miR-34a and miR-181a inhibition, so we can suppose the interaction between visfatin and the studied miRNA, via NF-κB pathway.

## 5. Conclusions

The present study adds new information about the existing interaction between visfatin and miR-34a and miR-181a in human OA chondrocyte cultures.

First of all, transfection experiments on our cultures demonstrate the involvement of miR-34a and miR-181a in the modulation of apoptosis and oxidative stress processes induced by visfatin. 

Then, the use of NF-κB specific inhibitor corroborates the implication of this pathway in the regulation of visfatin-mediated effects. Besides, the silencing of miR-34a and miR-181a counteracted NF-κB activation and translocation, leading us to suppose that the complex cross-talk between visfatin and the studied miRNA is mediated by NF-κB signalling pathway.

The results of this paper may provide insight into the underlying molecular mechanism by which visfatin induces apoptosis and oxidative stress on chondrocytes, probably through the modulation of miR-34a and miR-181a via NF-κB signalling pathway; these findings highlight the importance of visfatin as potential therapeutic target for the treatment of OA.

However, additional experiments on healthy primary cells are recommended to confirm the involvement of this pathway in mediating chondrocytes metabolism in OA pathogenesis. Further transfection experiments with a specific miRNA mimic could be useful for confirming the regulation induced by the studied miRNA, analysing the effects caused by miR-34a and miR-181a over-expression. Finally, a deeper investigation of NRF2 factor could be useful to better understand the role of visfatin and miRNA in the regulation of oxidant/antioxidant balance.

## Figures and Tables

**Table 1:** Primers used for real time PCR.

**miRNA Genes**	**Cat. No. (Qiagen)**
miR-34a	MS00003318
miR-181a	MS00006692
SNORD-25	MS00014007
**Target Genes**	**Cat. No. (Qiagen)**
BCL2	QT00000721
SOD-2	QT01008693
CAT	QT00079674
NRF2	QT00027384
ACTB	QT00095431

Abbreviations: miRNA = microRNA; SNORD-25 = Small Nucleolar RNA, C/D Box 25; BCL2 = B-cell lymphoma; SOD-2 = superoxide dismutase 2; CAT = catalase; NRF2 = nuclear factor erythroid 2 like 2; ACTB = Actin Beta.
